# A Meta-Analytic Study of the Neural Systems for Auditory Processing of Lexical Tones

**DOI:** 10.3389/fnhum.2017.00375

**Published:** 2017-07-26

**Authors:** Veronica P. Y. Kwok, Guo Dan, Kofi Yakpo, Stephen Matthews, Peter T. Fox, Ping Li, Li-Hai Tan

**Affiliations:** ^1^Center for Language and Brain, Shenzhen Institute of Neuroscience Shenzhen, China; ^2^Neuroimaging Laboratory, School of Biomedical Engineering, Shenzhen University Health Science Center Shenzhen, China; ^3^Guangdong Key Laboratory of Biomedical Information Detection and Ultrasound Imaging Shenzhen, China; ^4^Department of Linguistics, School of Humanities, University of Hong Kong Hong Kong, Hong Kong; ^5^Research Imaging Institute, University of Texas Health Science Center at San Antonio San Antonio, TX, United States; ^6^South Texas Veterans Health Care System San Antonio, TX, United States; ^7^Department of Psychology, and Center for Brain, Behavior, and Cognition, Pennsylvania State University University Park, PA, United States

**Keywords:** meta-analysis, lexical tones, auditory processing, neuroimaging, activation likelihood estimation (ALE) meta-analysis

## Abstract

The neural systems of lexical tone processing have been studied for many years. However, previous findings have been mixed with regard to the hemispheric specialization for the perception of linguistic pitch patterns in native speakers of tonal language. In this study, we performed two activation likelihood estimation (ALE) meta-analyses, one on neuroimaging studies of auditory processing of lexical tones in tonal languages (17 studies), and the other on auditory processing of lexical information in non-tonal languages as a control analysis for comparison (15 studies). The lexical tone ALE analysis showed significant brain activations in bilateral inferior prefrontal regions, bilateral superior temporal regions and the right caudate, while the control ALE analysis showed significant cortical activity in the left inferior frontal gyrus and left temporo-parietal regions. However, we failed to obtain significant differences from the contrast analysis between two auditory conditions, which might be caused by the limited number of studies available for comparison. Although the current study lacks evidence to argue for a lexical tone specific activation pattern, our results provide clues and directions for future investigations on this topic, more sophisticated methods are needed to explore this question in more depth as well.

## Introduction

The functional anatomy of speech perception has been intensively investigated for over a century in the neuropsychology literature, and more recently in the neuroimaging literature. Speech processing is known to preferentially rely on cortical regions in the left hemisphere (Hickok and Poeppel, 2007), but neural specialization of different aspects (e.g., tone, rhyme, stress and other spectral and temporal properties) of speech remains controversial. For tonal language speakers, lexical tone plays a critical role in spoken word recognition, which involves complex acoustic and phonological processes. While a large number of studies have been designed to uncover the perceptual and cognitive mechanisms in tone processing, it is only until recently that researchers have begun to focus on the neural substrates underlying tone perception. Since around half of the world’s languages are tonal (Maddieson, 2013), understanding how lexical tone is processed and represented in the brain could provide significant insights into mechanisms of speech perception.

Lexical tone in tonal languages is characterized by pitch variations at the syllable level, and it is used to distinguish lexical or grammatical meanings. While it is known that the processing of non-linguistic pitches such as music and melodies are associated with right hemispheric regions (e.g., Zatorre et al., 1992, 1994), researchers have started to ask whether the neural specialization for linguistic pitch patterns (i.e., lexical tones) would be different from that of non-linguistic pitch patterns. Two prominent models have been proposed regarding the hemispheric dominance of human pitch perception, the domain-specific model and the cue-specific model. The domain-specific model (or the functional hypothesis) assumes lateralization depends on the function of pitch patterns: when tones are processed as acoustic units (i.e., pure variation in pitch) their processing is right lateralized, but when they are processed as phonological units (i.e., as linguistic or semantic information) their processing is left lateralized (Whalen and Liberman, 1987; Liberman and Whalen, 2000). By contrast, the cue-specific model (or the acoustic hypothesis) assumes that pitch patterns are processed according to their acoustic structures, regardless of their functions, and are therefore lateralized only to the right hemisphere (Van Lancker, 1980; Zatorre and Belin, 2001).

The brain basis of lexical tone processing has been examined in neuroimaging studies for over a decade. East Asian tone languages such as Mandarin and Thai have been frequently studied in order to investigate neural correlates underlying lexical tone perception in native speakers of tonal language. In the brain imaging literature, the cortical representation of lexical tone perception has been examined with several approaches. Early studies have focused on the cross-linguistic comparisons of the neural basis for lexical tone perception in tonal vs. non-tonal language speakers (Gandour et al., 1998, 2000, 2002, 2003; Hsieh et al., 2001; Klein et al., 2001; Wong et al., 2004). These cross-linguistic studies have consistently indicated left hemisphere specialization for lexical tone processing in native speakers of tonal languages, contrasting the right hemisphere specialization in native speakers of non-tonal languages. Since non-tonal language speakers who have had no prior experience with a tonal language failed to show brain activity in the left hemisphere in lexical tone perception, Wang et al. (2003) and Wong et al. (2007) conducted lexical tone training studies to test whether American learners could process lexical tones in ways similar to native speakers after learning. They found that successful lexical tone learners showed enhanced cortical activations in left superior temporal regions (BA22, BA42), whereas less successful learners showed greater activation in the right hemispheric regions relative to the successful learners, such as the superior temporal region and inferior frontal gyrus. More recent studies have focused on examining the neural system of lexical tone perception in native tonal language speakers, with two commonly used experimental paradigms: explicit lexical tone perception (Li et al., 2003, 2010; Xu et al., 2006; Nan and Friederici, 2013; Yu et al., 2014) and lexical tone production (Liu et al., 2006; Chang et al., 2014). These investigations with native speakers have indicated contributions of numerous brain regions in the processing of lexical tones, including: (1) bilateral frontal language areas (i.e., posterior prefrontal gyrus, middle frontal gyrus); (2) bilateral fronto-parietal network; (3) bilateral superior temporal and surrounding regions; and (4) the left insular cortex. Moreover, structural imaging studies have reported increased gray matter volume in brain regions of tonal language speakers relative to non-tonal language speakers, such as the left Heschl’s gyrus (Wong et al., 2008), left insula/transverse temporal gyrus (BA42) and right superior temporal gyrus (Crinion et al., 2009).

Although previous studies have enhanced our understanding of the neural basis of auditory tone perception, these studies have generated markedly different patterns of findings and failed to show consistent patterns of hemispheric laterality of linguistic pitch processing in native speakers of tonal language. These divergent results are likely due to inter-subject variability and differences in experimental tasks, among other variables that characterize different studies. The present study is designed to analyze such variables across the existing neuroimaging studies of tone perception, aiming at providing a clearer picture of the brain networks that are most consistently involved in auditory perception of lexical tones. This study is exempt from ethics approval. In particular, we utilized a meta-analytic technique, activation likelihood estimation (ALE) method, to quantitatively synthesize results across published data from healthy adult participants in the relevant literature, and to reveal patterns of convergence among the brain regions associated with lexical tone perception. We did not recruit more human subjects for further analysis. Meta-analysis has proven to be an important statistical method to combine results from independently published brain imaging studies that may involve different task designs and scanner equipment. Integration of data from multiple studies could increase statistical power of findings, thereby providing a stronger conclusion than arguments gained from individual studies (Turkeltaub et al., 2012). However, a single meta-analysis might not be sufficient to explain the commonalities and specificities in the brain processing of lexical tones when compared to regular speech processing in general. Thus, we also conducted a meta-analysis that involved studies with similar language conditions in non-tonal languages, in which participants were engaged in lexico-semantic processing when auditory stimuli were presented. We sought to compare neural mechanisms engaged in both processes in tonal and non-tonal langauges, and to investigate the neural substrates specifically mediating lexical tone processing in tonal languages.

## Materials and Methods

### Literature Selection

We utilized the PubMed database^1^ to search for articles relevant to the meta-analysis. All selected lexical tone articles fulfilled the following selection criteria: (1) all involved normal, healthy, right-handed adults; (2) lexical tone related tasks were used in the studies (all involved task-activation paradigms); and (3) all reported imaging data on 3D coordinates (*x*, *y*, *z*) in stereotactic space. With these selection criteria, we found 24 neuroimaging studies of auditory lexical tone processing in the PubMed database (as of March 27, 2017). Among these 24 studies, we excluded three studies that measured and contrasted subjects’ structural brain volume (Wong et al., 2008; Crinion et al., 2009; Qi et al., 2015), because brain structural differences may not be directly associated with lexical tone processing, and in this meta-analysis we therefore focus only on the cortical activation evoked by functional tasks. One functional MRI study (Zhang et al., 2011) was also excluded, because it was based on region-of-interest (ROI) analyses while the ALE meta-analysis computes whole-brain analysis data only. We further excluded three studies that used silence/rest as baseline (Klein et al., 2001; Xu et al., 2006; Kwok et al., 2016), since these contrasts do not provide activation specific to language/auditory stimuli processing.

All selected articles for the control meta-analysis involved: (1) healthy, right-handed adults; (2) auditory lexical decision task was used in the studies (all involved task-activation paradigms); and (3) all reported imaging data on 3D coordinates (*x*, *y*, *z*) in stereotactic space. With these selection criteria, we found 19 neuroimaging studies involved in access to lexical information through audition in non-tonal languages in the PubMed database (as of March 27, 2017). Among these 19 studies, we excluded one study that did not state whether the 3D coordinates were in MNI or Talairach space (Roxbury et al., 2014), two more studies were excluded because they did not report the activation contrasts that we were interested in (Prabhakaran et al., 2006; Minicucci et al., 2013). We further excluded one study that used silence/rest as baseline (Zhuang et al., 2011), since these contrasts do not provide activation specific to language/auditory stimuli processing.

According to these inclusion and exclusion criteria, a set of 17 studies with Mandarin and Thai tone perception was entered into our meta-analysis: 11 used an explicit tone perception task (Gandour et al., 1998, 2000, 2002, 2003; Hsieh et al., 2001; Li et al., 2003, 2010; Wong et al., 2004; Nan and Friederici, 2013; Zhang et al., 2016, 2017), three used Mandarin tone production (Liu et al., 2006; Chang et al., 2014; Chang and Kuo, 2016), and three used Mandarin tone training (Wang et al., 2003; Wong et al., 2007; Yang et al., 2015). Among the 17 studies, 13 utilized fMRI, and four utilized positron emission tomography (PET) to acquire brain images. Among the set of 15 studies that involved subjects to engage in the access of lexical information through audition in non-tonal languages, all of them used auditory semantic/lexical decision task, 14 acquired the imaging data through fMRI and one used PET. Tables 1 and 2 present the full details of the selected studies. Although the studies had different baseline conditions due to the tasks used and issues addressed, the ALE meta-analysis of these data should allow us to determine the neural mechanisms subserving auditory lexical tone processing in tonal languages and auditory lexical processing in non-tonal languages.

**Table 1:** Summary of selected literature for lexical tone meta-analysis.

Study	Language	*N*	Experimental task	Baseline
Gandour et al. (1998)	Thai	5	Tonal discrimination	Nonspeech pitch discrimination
Gandour et al. (2000)	Thai	5	Tonal discrimination	Nonspeech pitch discrimination
Hsieh et al. (2001)	Mandarin	20	Tonal discrimination	Passive listening
Gandour et al. (2002)	Mandarin, Thai	20	Tonal discrimination	Nonspeech pitch discrmination
Gandour et al. (2003)	Mandarin	20	Tonal discrimination	Passive listening
Li et al. (2003)	Mandarin	12	Tonal discrimination	Syllable discrimination
Wang et al. (2003)	Mandarin	6	Tone identification	Visual, auditory and motor control tasks
Wong et al. (2004)	Mandarin	7	Tonal discrimination	Passive listening
Liu et al. (2006)	Mandarin	10	Pinyin-naming; character-naming	Fixation
Wong et al. (2007)	English	17	Tonal discrimination	Sinewave discrimination
Li et al. (2010)	Mandarin	12	Tonal discrimination	Consonant and vowel discrimination
Nan and Friederici (2013)	Mandarin	18	Tone congruity judgment of Chinese phrases	Tone congruity judgment of musical phrases
Chang et al. (2014)	Mandarin	15	Production of mixed tone sequences	Production of repeated tone sequences
Yang et al. (2015)	English	39	Tonal discrimination	Fixation
Chang and Kuo (2016)	Mandarin	30	Production of mixed tone sequences	Production of repeated tone sequences
Zhang et al. (2016)	Cantonese	19	Lexical tone discrimination	Talker’s voice discrimination
Zhang et al. (2017)	Cantonese	11	Lexical tone discrimination	Musical notes discrimination

**Table 2:** Summary of selected literature for auditory lexical judgment meta-analysis.

Study	Language	*N*	Experimental task	Baseline
Dapretto and Bookheimer (1999)	German	8	Semantic judgment	Syntactic judgment
Kotz et al. (2002)	German	13	Semantic task	Semantic judgment of nonwords
Rissman et al. (2003)	English	15	Semantic task	Semantic judgment of nonwords; tone decision
Poeppel et al. (2004)	English	10	Semantic task	Categorical perception; FM sweeps
Orfanidou et al. (2006)	English	13	Lexical decision of real words	Lexical decision of nonwords
Palti et al. (2007)	Hebrew	14	Semantic judgment	Semantic judgment of reversed words
Binder et al. (2008)	English	26	Semantic task	Non-speech tone decision; phoneme decision
Bilenko et al. (2009)	English	16	Semantic judgment of ambiguous words	Semantic judgment of unambiguous words
Raettig and Kotz (2008)	German	16	Semantic judgment of real words	Semantic judgment of nonwords
Ruff et al. (2008)	English	15	Semantic judgment	Lexical decision
Balthasar et al. (2011)	German	18	Homonym decision	Target word decision
Wright et al. (2011)	English	14	Lesical decision task of real words	Non-speech tone decision
Méndez Orellana et al. (2014)	Dutch	20	Semantic task	Presentation of nonwords
Lopes et al. (2016)	Brazilian	24	Semantic decision	Non-speech tone decision
Ludersdorfer et al. (2016)	German	29	Semantic task	Tone decision

### Activation Likelihood Estimation (ALE)

The GingerALE software package is available on BrainMap website^2^. ALE is a coordinate-based meta-analysis technique that assesses the convergence of activation foci from different neuroimaging studies, modeled as probability distributions of activation at given coordinates against null distributions of random spatial associations between studies (Turkeltaub et al., 2012; Laird et al., 2005; see Wager et al., 2007 for review). The method has been used widely in recent years as an effective meta-analysis tool for functional imaging data.

The reported tasks involved auditory lexical tone judgment (266 subjects, 160 activation foci) and auditory lexical semantic judgment (251 subjects, 259 activation foci), in which participants were instructed to make discrimination judgments to the presented lexical tones. The activation foci generated in the contrasts of lexical tone tasks relative to baseline tasks (i.e., passive listening, non-speech pitch discrimination) and the contrasts of auditory lexical decision relative to baseline tasks (i.e., non-word judgment, non-speech tone decision) were included in the analysis, all foci data was imported to a text file and entered into the ALE software.

ALE maps were computed for 17 auditory lexical tone studies and 15 auditory lexical decision studies respectively. Prior to the analysis, all coordinates were transformed into a single stereotactic space: all MNI coordinates were converted into Talairach space using the icbm2tal transform tool (Lancaster et al., 2007) implemented in GingerALE software package (Eickhoff et al., 2009, 2011). ALE maps were generated by the ALE method (Turkeltaub et al., 2012), using a full-width half-maximum (FWHM) of 10 mm. Each reported coordinate was treated as the center for the 3D Gaussian probability distribution. Statistical significance was determined by a permutation test of randomly distributed foci. The test was corrected for multiple comparisons using the false discovery rate (FDR) with a threshold at *P* < 0.05 corrected, with 100 mm^3^ minimum volume size.

## Results

**Figure 1:**
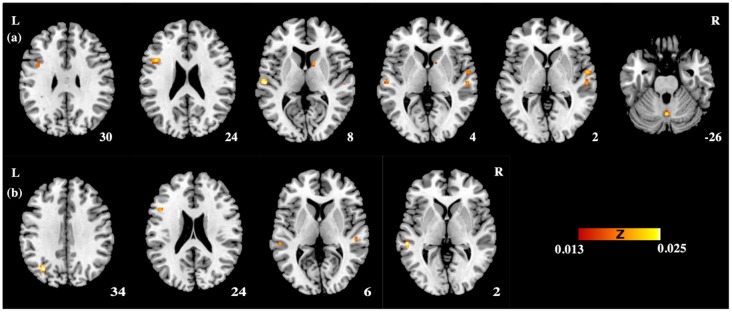
The activation likelihood estimation (ALE) maps showing significant activation likelihood across studies of **(A)** auditory processing of lexical tones (*P* < 0.05 false discovery rate (FDR) corrected); **(B)** auditory lexico-semantic processing in non-tonal languages (*P* < 0.05 FDR corrected). L, left hemisphere; R, right hemisphere.

**Figure 2:**
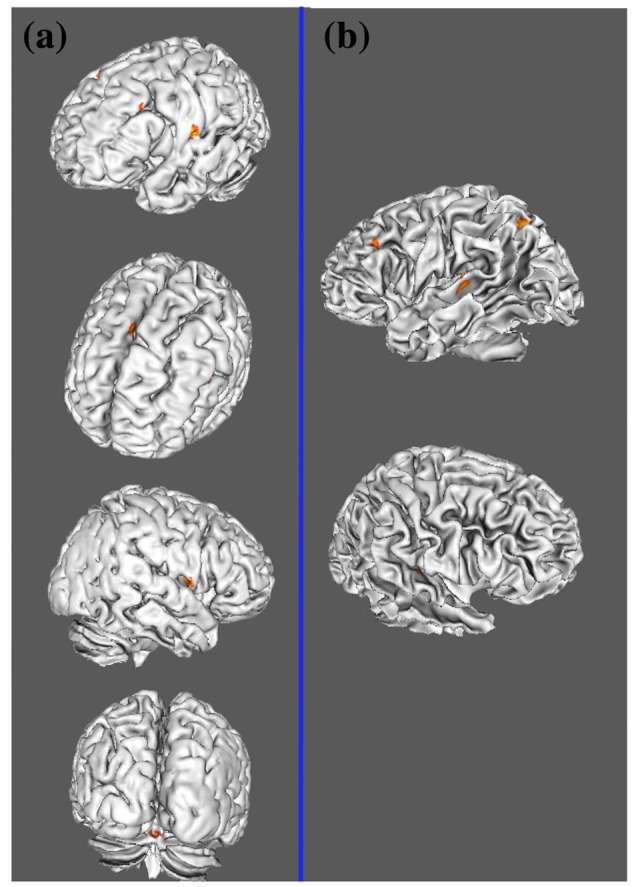
Lateral view of two meta-analyses. **(A)** ALE results of auditory lexical tone processing in tonal languages; **(B)** ALE results of auditory lexico-semantic processing in non-tonal languages.

Table 3 and Figures 1A, 2A illustrate the results of our ALE meta-analysis of the selected literature on auditory lexical tone processing. Eight clusters of activation likelihood are identified. First, the left inferior frontal cortex with two submaxima located in left inferior frontal gyrus (BA44; *x* = −44, *y* = 12, *z* = 24; BA9; *x* = −40, *y* = 4, *z* = 30), and the right medial frontal gyrus (BA8; *x* = 2, *y* = 18, *z* = 44) play central roles in auditory lexical tone processing. The left prefrontal cortex shows the highest convergence, with cluster sizes of 760 and 552 mm^3^ respectively. Second, several other brain regions are consistently involved in mediating lexical tone perception, including the left posterior transverse temporal gyrus (BA42; *x* = −58, *y* = −18, *z* = 8), right superior temporal gyrus (BA 22; *x* = 58, *y* = −6, *z* = 2). Third, although their activation clusters are around 300 mm^3^ or below, the right anterior cerebellum (*x* = 2, *y* = −64, *z* = −26), right transverse temporal gyrus (BA41; *x* = 56, *y* = −22, *z* = 4), and right caudate (*x* = 12, *y* = 6, *z* = 8) may also be implicated in lexical tone processing.

**Table 3:** Activation likelihood estimation (ALE) meta-analysis of auditory processing of lexical tones*.

Anatomical region	BA	Coordinates			ALE (×10^−2^)	Volume (mm^3^)
		*x*	*y*	*z*		
L inferior frontal gyrus	44	−44	12	24	1.84	760
	9	−40	4	30	1.49	
R medial frontal gyrus	8	2	18	44	1.84	552
L posterior transverse temporal gyrus	42	−58	−18	8	2.21	544
R superior temporal gurys	22	58	−6	2	1.84	320
R anterior cerebellum	-	2	−64	−26	1.88	312
R posterior transverse temporal gyrus	41	56	−22	4	1.82	280
R caudate	-	12	6	8	1.66	136

*BA, Brodmann area; L, left; R, right. Coordinates were based on Talairach space.

Table 4 and Figures 1B, 2B illustrate the results of our ALE meta-analysis of the selected literature on auditory lexical processing in non-tonal languages. Four clusters of activation likelihood are identified. The left precuneus (BA19; *x* = −34, *y* = −68, *z* = 34) shows the largest convergence with a cluster size of 496 mm^3^, followed by the left middle temporal gyrus (BA22; *x* = −56, *y* = −34, *z* = 2; cluster size: 400 mm^3^). The left inferior frontal gyrus (BA9; *x* = −46, *y* = 16, *z* = 22) and right superior temporal gyrus (BA41; *x* = 52, *y* = −26, *z* = 6) have relative small cluster sizes below 200 mm^3^, but these brain regions are activated when subjects were accessing lexical semantics through an auditory paradigm in non-tonal languages.

**Table 4:** ALE meta-analysis of auditory lexical decision in non-tonal languages*.

Anatomical region	BA	Coordinates			ALE (×10^−2^)	Volume (mm^3^)
		*x*	*y*	*z*		
L precuneus	19	−34	−68	34	2.52	496
L middle temporal gyrus	22	−56	−34	2	2.29	400
L inferior frontal gyrus	9	−46	16	22	1.99	168
R transverse temporal gyrus	41	52	−26	6	1.78	112

*BA, Brodmann area; L, left; R, right. Coordinates were based on Talairach space.

### Contrast Analysis

Next, we performed a contrast analysis to investigate neural correlates, which were more specific to lexical tone processing in tonal languages relative to the processing of lexical semantics through audition in non-tonal languages. Yet, no significant differences were found. The opposite contrast (i.e., non-tonal relative to tonal) also showed no significant differences as well. The only significant result was obtained from the conjunction analysis. The cortical activation in the left inferior frontal gyrus (BA9; *x* = −46, *y* = 14, *z* = 24; cluster size = 16 mm^3^) showed significant similarity between both datasets. According to the GingerALE software, at least 15 studies in each dataset are required in order to have enough statistical power (Cortese et al., 2012; Wagner et al., 2014). In this study, we only have marginally sufficient number of studies in each dataset (17 and 15 articles, respectively) and thus, the failure to reach significance might be due to the lack of statistical power. Since both datasets contained only limited number of studies, we further analyzed the data in a more lenient approach, at *P* < 0.001 and *P* < 0.005 uncorrected threshold (see Table 5). No cluster survived in the tonal vs. non-tonal contrast at both thresholds, but we found activations in the opposite contrast (i.e., non-tonal vs. tonal). At uncorrected *P* < 0.001, one cluster of activation likelihood is identified at the left middle temporal gyrus (BA19; *x* = −43, *y* = −73, *z* = 26; BA39; *x* = −40, *y* = −68, *z* = 27) with a cluster size of 952 mm^3^. When the threshold further dropped to uncorrected *P* < 0.005 for non-tonal vs. tonal contrast, three clusters of activation likelihood are identified with cluster sizes of 2384, 1416 and 208 mm^3^, respectively. All clusters are located in the left temporo-parietal area, including left middle temporal gyrus (BA19; *x* = −43, *y* = −73, *z* = 26; BA39; *x* = −38, *y* = −71, *z* = 28; BA21; *x* = −54, *y* = −32, *z* = −8), left angular gyrus (BA39; *x* = −48, *y* = −60, *z* = 34), left superior occipital gyrus (BA19; *x* = −40, *y* = −78, *z* = 32) and the left superior temporal gyrus (BA38; *x* = −50, *y* = 6, *z* = −10).

**Table 5:** ALE contrast analysis of non-tonal vs. tonal auditory processing*.

Anatomical region	BA	Coordinates			ALE	Volume (mm^3^)
		*x*	*y*	*z*		
At *p* < 0.001 uncorrected
L middle temporal gyrus	19	−43	−73	26	3.24	952
	39	−40	−68	27	3.09	
At *p* < 0.005 uncorrected
L middle temporal gyrus	19	−43	−73	26	3.04	2384
	39	−38	−71	28	2.88	
L angular gyrus	39	−48	−60	34	2.75	
	39	−52	−65	35	2.73	
L superior occipital gyrus	19	−40	−78	32	2.71	
L middle temporal gyrus	21	−54	−32	−8	3.04	1416
	21	−58	−35	3	2.99	
	21	−57	35	−3	2.95	
	21	−59	−40	0	2.88	
L superior temporal gyrus	38	−50	6	−10	3.16	208
	21	−51	4	−14	3.04	
L middle temporal gyrus	21	−52	2	−18	2.77

*BA, Brodmann area; L, left. Coordinates were based on Talairach space.

## Discussion

There has been a growing literature in the auditory processing of lexical tones from a neurocognitive perspective, as seen in the number of publications devoted to this subject in the last decade (for a review, see Gandour, 2006). Given the importance of tones in the speech perception of languages such as Chinese and Thai, it is important for us to understand the neurocognitive mechanisms underlying lexical tone perception. However, there has been no consensus on the specific brain regions that support this perception or the overall lateralization pattern that subserves the process. In this study, we performed an ALE meta-analysis on the growing literature in the neuroimaging study of tone perception. In order to gain in-depth understanding on the neural systems specific to the processing of lexical tones, we performed a control ALE meta-analysis on a similar language condition in non-tonal languages for the sake of comparison. The results of the present ALE meta-analyses shed light on the neural basis of lexical tone processing in speech. Both analyses reveal that activation clusters are centered at the frontal and temporal regions, highlighting the importance of the inferior prefrontal and superior temporal regions for speech processing (Hickok and Poeppel, 2007).

The ALE results of auditory lexical tone processing showed that the largest cluster with the highest convergence was located in the left inferior prefrontal gyrus (i.e., BA44 and BA9). The left PFC has been consistently associated with the extraction of phonetic information, such as extraction of consonant structure (Zatorre et al., 1992, 1996; Binder et al., 1997; Burton, 2001). Apart from pitch processing, the inferior frontal gyrus has also been implicated in lexical-semantic processing (Petersen et al., 1988; Rumsey et al., 1997; Mummery et al., 1999; Tan et al., 2001; Chan et al., 2004). Thus, this region is also activated in our control meta-analysis on non-tonal languages. The major function of lexical tones in tonal languages such as Chinese and Thai serves to distinguish meanings, and therefore we assume that the prefrontal cortex is responsible for processing both the lexical pitch and lexical semantics of auditory linguistic stimuli.

The second largest activation cluster was located in the right medial frontal gyrus (BA8). Previous studies have revealed several functions of this brain region. The right medial frontal cortex is responsible for maintaining memory and attention, and is highly important for executive function tasks (Simons and Spiers, 2003; Baird et al., 2006; Euston et al., 2012). This region is also associated with pitch perception, such as tonality processing (Janata et al., 2002) and pitch identification among non-musicians (Schwenzer and Mathiak, 2011). Moreover, it is involved in the spectral processing of acoustic signals (Pedersen et al., 2000; Reiterer et al., 2005). Since we did not found any significant activation in the medial frontal gyrus in our control meta-analysis, we assume that this region plays a crucial role in processing lexical tone information.

The left posterior transverse temporal area (BA42) was the third largest activation cluster. Our analysis shows that the bilateral transverse temporal gyrus (BA41, 42) are consistently involved in lexical tone processing, although the level of convergence in the right transverse temporal gyrus was relatively lower than that in the left hemisphere. However, BA41 and 42 were not involved in the auditory lexical processing in non-tonal languages. Previous studies have indicated that the basic processing of simple acoustic stimuli, such as frequency-modulated tones and sound with discontinuous acoustic patterns, activate BA42 (Mirz et al., 1999; Binder et al., 2000). Thus, we hypothesize that the transverse temporal region is involved in the initial processing of auditory stimuli that may not be speech-related. The right superior temporal gyrus (STG; BA22) was also involved in mediating auditory processing of lexical tones according to our analysis. The right STG has been repeatedly shown to be critical to perceptual pitch processing, vocal pitch error detection and voice control in the literature (Robin et al., 1990; Johnsrude et al., 2000; Zatorre and Belin, 2001; Zatorre et al., 2002; Flagmeier et al., 2014), and in the case of Chinese tones, shows more sensitivity to acoustic than phonological variations (Zhang et al., 2010, 2011).

A final region showing significant activation in our analysis was the right caudate. The caudate is involved in various motor and non-motor processes (Seger and Cincotta, 2005; Grahn et al., 2008). Some suggested that the caudate might be the center of language control (Friederici, 2006), and involved in selection or inhibition of language (Robles et al., 2005; Wang et al., 2007). The lexical tone discrimination tasks mostly required subjects to distinguish lexical tone information only, while subjects actually processed both phonology and meaning of the presented syllable as a whole at the same time. Thus, the caudate seems to participate in suppressing further analysis on the vowel, consonant or the lexico-semantics but focus on the extraction of lexical tone information.

Since both meta-analyses examined the processing of lexical information through audition, some common cortical regions are activated, such as the left inferior frontal gyrus (BA9) and bilateral superior temporal regions. When looking at the results of two individual ALEs, relative to lexico-semantic processing of non-tonal languages, lexical tone processing in tonal languages appeared to recruit more right hemispheric regions such as the medial frontal gyrus, right transverse temporal gyrus, right caudate and right anterior cerebellum. However, our results in the contrast analysis could not support this argument. Moreover, it is apparent that most lesion evidence suggests left hemispheric dominance in lexical tone processing in tonal languages (Naeser and Chan, 1980; Gandour and Dardarananda, 1983; Hughes et al., 1983; Packard, 1986).

One major limitation of this study is the insufficient number of studies available for a powerful contrast analysis between datasets that we are interested in. Thus, we lack evidence to make a strong and convincing claim on the lexical tone specific neural network, if there is any. More sophisticated methods are required to investigate the specificity in lexical tone processing. Despite our failure to obtain significant results from the contrast analysis, we tried to visualize the trend of the potential differences between tonal and non-tonal auditory processing at less stringent statistical thresholds (*P* < 0.001, *P* < 0.005 uncorrected for multiple comparisons). When compared to tonal processing, the left temporo-parietal regions are more activated in non-tonal processing, mainly in the left middle temporal gyrus and the left angular gyrus (BA19 and 39). The left middle temporal gyrus and left angular gyrus have been implicated in lexical semantic processing, and according to the dual-stream model of speech processing (Hickok and Poeppel, 2000, 2004, 2007), these two regions are reliably activated across a range of semantic tasks (Démonet et al., 1992; Vigneau et al., 2006; Mashal et al., 2008; Binder et al., 2009; Seghier et al., 2010; Zhao et al., 2013). This finding showed that the temporo-parietal region is tended to be less involved in semantic processing in tonal languages. Future studies could focus on the investigation of semantic processing in lexical tones.

Apart from providing the overall picture across the 17 lexical tone studies, our lexical tone meta-analysis also offers insights into language processing across modalities. A recently published study on the neural basis of lexical tone reading has shown that lexical tone processing in reading Chinese characters involved a distributed network in both hemispheres including bilateral frontal regions, left inferior parietal lobule, left posterior middle/medial temporal gyrus, left inferior temporal region, bilateral visual systems and cerebellum (Kwok et al., 2015). In contrast to our analysis here that shows the crucial role of both the left and right STG, Kwok et al.’s (2015) lexical tone reading task involved no bilateral STG activation. Thus, our ALE results along with Kwok et al.’s (2015) data suggest that the bilateral STG are modality-specific regions for lexical tone perception in the spoken language only (see also Zhang et al., 2011).

In sum, our ALE results give a picture of the crucial brain regions processing non-tonal auditory lexicon and lexical tones respectively. Although we failed to uncover any lexical tone specific pattern at the moment, our findings provide valuable insights and directions to future investigations on tonal and non-tonal auditory processing, and more sophisticated methods are needed to explore this question in more depth.

## Author Contributions

VPYK, GD, PTF and L-HT designed and performed the research; VPYK, GD, KY, SM and L-HT analyzed the data; and VPYK, GD, PL and L-HT wrote the article.

## Conflict of Interest Statement

The authors declare that the research was conducted in the absence of any commercial or financial relationships that could be construed as a potential conflict of interest.

## Footnotes

^1^https://www.ncbi.nlm.nih.gov/pubmed

^2^www.brainmap.org/ale
